# Enhanced Mechanical and Thermal Properties of Stereolithography 3D Printed Structures by the Effects of Incorporated Controllably Annealed Anatase TiO_2_ Nanoparticles

**DOI:** 10.3390/nano10010079

**Published:** 2020-01-01

**Authors:** Suhail Mubarak, Duraisami Dhamodharan, Nidhin Divakaran, Manoj B. Kale, T. Senthil, Lixin Wu, Jianlei Wang

**Affiliations:** 1 CAS Key Laboratory of Design and Assembly of Functional Nanostructures, Fujian Key Laboratory of Nanomaterials, Fujian Institute of Research on the Structure of Matter, Chinese Academy of Sciences, Fuzhou 350002, China; suhail@fjirsm.ac.cn (S.M.); duraisamidhamodharan@fjirsm.ac.cn (D.D.); nidhin@fjirsm.ac.cn (N.D.); manojkale@fjirsm.ac.cn (M.B.K.); 2 University of Chinese Academy of Sciences, Beijing 100049, China; 3 Advanced Research School for Technology and Product Simulation, Central Institute of Plastics Engineering and Technology, Chennai 600032, India; tsenthilsci@gmail.com

**Keywords:** Thermal properties, Mechanical properties, Stereolithography, Thermal conductivity, 3D printing

## Abstract

Fabrication of low-cost, durable and efficient metal oxide nanocomposites were successfully synthesized and reinforced with photo-resin via 3-dimensional printing. Here, we put forward a novel approach to enhance the mechanical and thermal behaviors of stereolithography (SLA) 3D printed architecture by adding TiO_2_ nanoparticles (TNPs) in different crystalline phases (anatase and rutile), which were obtained at different annealing temperatures from 400 °C to 1000°C. The heat-treated anatase TNPs were scrutinized by X-ray diffraction(XRD), X-ray photoelectron spectroscopy (XPS), Raman spectroscopy, diffusive reflectance spectroscopy (DRS), and transmission electron microscopy (TEM) analysis. Among all the samples, at 800 °C, annealed anatase TNPs exposed a highly crystalline anatase phase, having a low energy bandgap and a comparably high tensile strength (47.43 MPa) and high elastic modulus (2.261 GPa) for the 3D printed samples, showing improvement by 103% and 32%, respectively, compared with the printed pristine stereolithography resin (SLR) sample. Moreover, enhanced storage modulus and tan *δ* values were achieved via the better interfacial interactions between the incorporated nanofillers and the SLR matrix. In addition to this, enhanced thermal conductivity and thermal stability of the SLR matrix were also noted. The low energy bandgap and nanoscale size of the fillers helped to achieve good dispersion and allowed the UV light to penetrate at a maximum depth through the photo resin.

## 1. Introduction

Over the past few decades, additive manufacturing (3D printing) technology has shown a remarkable growth, which promises enormous potentialities and has gained a lot of interest from a diverse range of fields such as biomedical science and engineering [1,2,3], printing electronics [4], microfluidics [5], and aerospace composites [6]. 3D printing technology demonstrates the fabrication process, which facilitates the layer-by-layer structure formation from computer-aided design (CAD) data [7,8]. Among all 3D printing technologies, stereolithography (SLA) technology has been noted as the first patented and promoted technique. Photopolymerization is the critical mechanism of SLA 3D printing technology, demonstrating superior performance in the manufacture of 3D objects with accuracy, rapid curing, and micro-nanoscale resolution [9,10,11]. Nowadays, the photopolymerization applications and their utilization among industries are striking as well as modest schemes involving polymer chemistry [12]. The working principle of SLA techniques has made it more conceivable to decrease the cost of the manufacturing processes with high-quality products [13]. Therefore, photopolymerization has gained outstanding attraction from researchers for its numerous engineering applications such as coatings, printing technologies, dental fillings, and the building of 3D structures [14,15,16]. The SLA process consists of the transformation of multifunctional urethanes or acrylic-based monomers and oligomers into an interconnected polymer by a propagation reaction initiated by free radicals or cations formed by the light source [17]. In general, most monomers and oligomers are unable to generate initiating species upon light exposure, which means that monomers and oligomers are indispensable to acquaint with some initiators that will assist in initiating polymerization through a photochemical reaction [18,19,20].

Conversely, fast curing and worthy spatial resolution benefit this technique, but fragility and poor bearing resistance leads to the serious drawbacks caused by non-uniform polymerization and poor cross-linking density, limiting the application of SLA printing [21,22]. Conversely, oxygen inhibition during free radical formation in the polymerization process has been a highly challenging phenomenon within photopolymerization techniques. Numerous processes have been exploited to lessen or completely conquer oxygen inhibition trouble in free radical polymerizations [23]. Sajjad-Dadashi et al. showed that ZnO nanoparticles can initiate the polymerization of vinyl monomers such as methyl methacrylate in organic systems in the presence of O_2_ under ultra violet (UV) light. ZnO or Fe/ZnO nanoparticles can recruit O_2_ in the initiation mechanism to prevent inhibition of the initiating radicals [24]. Additives such as amines and silanes that consume dissolved O_2_ in the system are the most common ways of preventing oxygen inhibition [25,26]. In addition, weak mechanical properties and deprived accuracy of stereolithography resin (SLR) are widespread problems for most desktop-level SLA techniques, restricting their utilization as functional materials [27]. For this reason, the development of mechanical behaviors of SLRs has been given attention in their applications [28,29]. To overcome this critical issue, many forms of research have been conducted, such as pre-treatment of SLA resin and incorporation of nanoparticles. Among all the techniques, the incorporation of nanoparticles achieves a better response over SLA 3D printing. The literature report evidently proves that the mechanical strength and thermal stability of 3D printed parts could be intrinsically enhanced by introducing nanofillers to the photopolymer matrix [30]. In accordance to the loaded nanoparticles, concordance with the resin could be a vital factor in making excellent interactions, leading to the enhancement of interlayer bonding, which would lead to the superior load allocation to form the polymer matrix [31,32]. Lately, the materialization of nanocomposites through enhancing photopolymerization has engrossed a huge amount of attention of researchers. In particular, there is a vast consideration based on the nano distribution phases comprising nanosized metals [33,34,35,36,37,38,39]. The metal oxide-based semiconductor nanoparticle has been the focus of substantial research attention in recent years [40]. Several efforts have been made in the preparation of these nanoparticles, and their broad applications vacillate in the field of photovoltaic cells, optoelectronics, and biosensors for environmental applications such as water purification and dangerous waste refinement [41,42,43,44,45].

Among the diverse nanoparticles, titanium dioxide has been comprehensively explored, owing to its attractive mechanical behaviors and prospective technological coverings as reinforcing nanofillers in an extensive variety of polymers. Conversely, the eco-friendly, high refractive index, optical transparency, low toxicity, low-cost, and high mechanical behaviors of titanium dioxide nanoparticles (TNPs) are thought to make it one of the greatest effective semiconductor photocatalysts for conservational applications. In addition, the low energy bandgap of about 3.2 eV of TNPs stimulates excellent photocatalysis reactions under UV light irradiation [46,47,48,49,50]. Nowadays, many nanostructured TiO_2_ materials, such as nanoparticles, nanotubes, and nanowires, are synthesized using many proposed routes such as sol-gel, hydrothermal and anodic oxidation methods [51,52,53,54,55,56,57,58]. These photocatalytic activities of commercial titanium dioxide (TiO_2_) are extensively used for the photodegradation of organic pollutants and dyes [59,60]. The dye degradation process is followed by the formation of active radical species resulting from the reduction of molecular oxygen (O_2_) by photostimulated electron-hole pairs [61,62,63,64]. In addition to the TNPs, other inorganic semiconductors nanoparticles, such as ZnO and Fe_2_O_3_, have also been performed as free-radical photoinitiators for curing photo resins, due to their proficiency of producing electron-hole pairs that assist the generation and mobility of reactive species to enhance photopolymerization [65,66].

Herein, this work emphasizes the generosity of innovative harmonization of nanotechnology and 3D printing technology, which have led to a technological revolution in this era, producing vast promises in science and engineering. In the present work, we synthesized nanosized anatase TiO_2_ particles via sol-gel process followed by treatment of different calcinations to develop a novel initiator for the photopolymerization of acrylic-urethane resin under UV irradiation. From our research study, it was revealed that these heat-treated anatase TNPs expose different crystalline and phase (anatase and rutile) transitions during different calcinations temperatures. There are many research studies that have reported that the bandgap tuning effects are due to vacancy cluster transformation within the TiO_2_ structures [67,68,69,70]. However, there are no works reported that involve the bandgap tuning of TiO_2_ nanoparticles through studying the assistance of temperature. Here, we report a novel study approach, showing that treatment by different temperature-assisted anatase TiO_2_ nanoparticles can initiate as well as enhance the photopolymerization of methyl methacrylate (MMA) and polyurethane in organic systems in the presence of atmospheric O_2_ under UV light irradiation. Also, the introduced TNPs can recruit O_2_ in the polymerization mechanism to avert inhibition of the initiating free radicals. In addition, these incorporated TNPs can be easily detached from the polymer combination and reprocessed without any substantial deterioration in the photocatalysis activity. The different temperature-assisted semiconducting TNPs underwent various phase transformations, bandgap energies, and crystalline sizes. Conversely, the nano-based incorporated TNPs helped in achieving better dispersion and allowed the UV lights to penetrate to maximum depth through the resin, which led to enhancement in the photopolymerization process. However, the anatase TNPs that incorporated SLA 3D printed objects revealed better mechanical and thermal properties through the enhanced photopolymerization by the achievement of excellent dispersion of introduced nanofillers within the polymer matrix.

## 2. Experimental Section

### 2.1. Materials

Titanium tetrachloride (GR, 99.5%), acrylate-based UV curable resin, which consists of hydroxyl ethyl methacrylate (HEMA), hydroxyl propyl methacrylate (HPMA), and the photoinitiator 2,4,6-trimethyl benzoyl diphenyl phosphine oxide (TPO) were purchased from Macklin, (Shanghai, China). Ethanol (AR, anhydrous, 99.7%) was obtained from Sigma-Aldrich (Wuxi, China). The monomers CN9010, CN991, and SR209 were supplied by Sartomer Americas, (Exton, PA, USA), and this has been taken into account throughout the research work. 3-Glycidoxypropyl trimethoxysilane (KH570) was obtained from Wenhua chemicals (Shanghai, China). All other chemicals used for this research were analytical grade, and double-distilled water was utilized throughout the research.

### 2.2. Methods

#### 2.2.1. Synthesis of Anatase TNPs and Calcinations at the Different Temperatures

**Figure 1:**
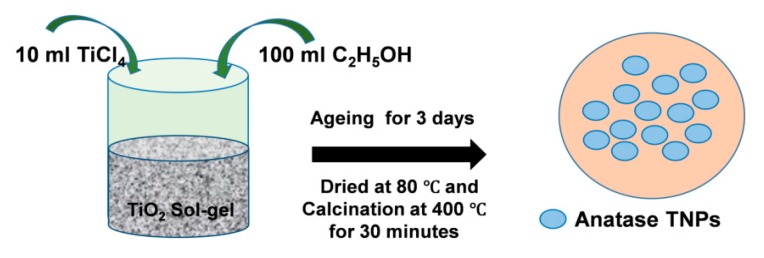
A detailed schematic route of anataseTiO_2_ nanoparticle (TNP) preparation by sol-gel method.

Anatase TiO_2_ nanoparticles were synthesized by the sol-gel method approach [71]. A total of 10 mL TiCl_4_ was slowly added dropwise to 100 mL of absolute ethyl alcohol at room temperature. A significant amount of HCl gas was exhausted for the period of the mixing process. A light yellow solution was acquired and accumulated for 3 days to form a gel form of TiO_2_ nanoparticles. Then, this solution was dehydrated at 80 °C until a dry powder was achieved. The dry whitish powder was annealed at 400 °C with a heating rate of 5 °C/min for 30 min. For studying the effect of annealing temperature on the thermal and mechanical behavior of as-prepared anatase TNPs, the synthesized anatase TNPs were heat-treated at 400 °C, 500 °C, 600 °C, 700 °C, 800 °C, 900 °C, and 1000 °C in a hot furnace at a heating rate of 5 °C/min for 1 h. The samples were named for abbreviation as ANT, with the suffix denoting the heat-treated temperature. For example, ANT400 was the anatase TNP treated at 400 °C, and so on. Figure 1 represents the schematic demonstration of the detailed synthesis process of anatase TNPs using the sol-gel method.

#### 2.2.2. Preparation of Nanocomposites for SLA 3D Printing.

To prepare the anatase TNP-incorporated SLA resin, a calculated amount of anatase TNPs were annealed at different temperatures, followed by the treatment with coupling agent KH570 (1% w/w of anatase TNPs) in an ethanol solution and dried at 80 °C in a blast oven for 2 h. To achieve better dispersion of anatase TNPs (nanofillers) within the SLA resin, the optimized amount of nanofillers were introduced into the SLA resin and this setup was maintained at vigorous stirring for 45 min at 70 °C [72]. An ultrasonication technique was also utilized to achieve better dispersion of fabricated nanoparticles before the process of 3D printing. The loading of nanofillers in the following description of this work was expressed as the weight (g) of added anatase TNPs per weight (g) of SLR matrix (denoted by % w/w). The as-prepared samples were named SLR for the neat photo resin and R/ANT400, R/ANT500, R/ANT600, R/ANT700, R/ANT800, R/ANT900, and R/ANT1000, representing the afore mentioned ANT400, ANT500, ANT600, ANT700, ANT800, ANT900, and ANT1000 nanoparticles, respectively, added to the stereolithography photo resin. Without specification in this work, the loading content of nanofillers with the photo resin was fixed at 1% w/w.

#### 2.2.3. Fabrication of Three-Dimensional Structures by SLA 3D Printer

In this project, the performed samples were printed by a UV laser irradiation-assisted Dream 3D-C200 stereolithography 3D printer to prepare all 3D printed specimens. The wavelength and intensity of the UV irradiation was at 405 nm and 155 mW/cm^2^, respectively was maintained, throughout the research study. The printer, which was utilized throughout research work, is displayed in Supplementary Figure S1. All the performed samples were American Society for Testing and Materials (ASTM) standard D638. After printing, the samples were further treated and washed by isopropanol for several times and post-cured in the presence of UV rays for 20 min.

### 2.3. Measurements and Characterization

#### 2.3.1. X-ray Diffraction (XRD) Measurements

The microstructures and crystalline phases of anatase TNPs were determined by low angle XRD (STOE-STADV diffractometer, STOE Corporation, Chicago, USA) using Cu-Kα radiation (λ= 1.59041 Å).

#### 2.3.2. Raman Spectroscopy Analysis

Raman spectrum of annealed anatase TNPs was determined with the assistance of a Raman Microscope (Renishaw, Invia, New Mills, UK) with a laser excitation wavelength of 537 nm.

#### 2.3.3. X-ray Photoelectron Spectroscopy (XPS) Analysis

The surface composition of elements and electronic states of the anatase TNPs were investigated by using X-ray photon spectroscopy (Thermo Scientific Escalab 250Xi, Waltham, MA, USA).

#### 2.3.4. FTIR Spectroscopy

A PerkinElmer spectrophotometer (Wellesley, MA, USA) equipment was determined to approve the IR spectrum of SLA resin and annealed anatase TNPs, with an average scanning rate of 64 scans and arrange varying from 4000 to 400 cm^−1^.

#### 2.3.5. Electron Microscope Analysis

High-resolution transmission electron microscopy (HRTEM)(JEOL JEM-2010, Tokyo, Japan) was engaged to perceive the morphologies of the ultrathin sectioned samples of as-prepared anatase TNPs, and selected area diffraction patterns (SAED) were identified at an acceleration potential of 200 kV. Field emission scanning electron microscope (FESEM) (JSM-7500F, SU8010/EDX, and Japan) was engaged to observe the morphologies of anatase TNPs and the fractured surfaces of 3D printed samples after performing the tensile study analysis.

#### 2.3.6. Diffusive Reflectance Spectroscopy (DRS)

The wavelength of the light absorbed by the material and its energy band gap was measured using diffusive reflectance spectroscopy (DRS) (Perkin Elmer Lambda 950 UV/VIS/NIR spectroscopy, Borken, Germany), and the energy bandgap was measured using a mathematical approach of Kubelka-Munk function.

#### 2.3.7. UV Rheological Analysis

A UV-LED accessory of DHR-2 (Discovery Hybrid Rheometer-2, TA instrument, New Castle, DE, USA) was used to examine the rheological study of neat SLA resin and SLR/nanofillers resin. The upper and lower geometry were prepared by aluminum and a transparent polymethyl methacrylate (PMMA), respectively, with a diameter of 20 mm each. The testing was performed for 100 s, and the light source was switched on at the 30th second of the testing irradiation intensity 155 mW/cm^2^.

#### 2.3.8. Mechanical Properties

The required mechanical properties were analyzed with the help of a universal material testing machine (AGX-100 plus, Shimadzu, Japan). The samples (length 60 mm, preferred thickness 2 mm, gauge distance 45 mm) were tested at a strain rate of 5 mm/min, and the ASTM method was followed for analysis. The five sets of each sample were considered for mechanical testing.

#### 2.3.9. Nanoindentation Test

The nanoindentation test was carried out on a nanoindenter instrument (Hysitron Inc., Tribo Indenter 750, Minneapolis, MN, USA) with a three-sided pyramid Berkovich diamond indenter. The diameter of the indenter probe was 100 nm. A matrix with 4 × 4 indentations was conducted on each sample, and the distance between two corresponding indentations was set as 10 µm.

#### 2.3.10. Dynamic Mechanical Properties

Dynamic mechanical analysis (DMA) data on samples were collected through the DMA Q800 System of TA Instruments using double cantilever performed from 25 °C to 180 °C at 5 °C/min at the frequency range of 1 Hz. The storage modulus and tangent delta (tan *δ*) for all the samples were recorded on the concerning temperature.

#### 2.3.11. Thermal Gravimetric Analysis (TGA)

Thermal gravimetric analysis was carried out using a TA Instrument. STA449C (New Castle, DE, USA) was custom to examine the thermal stability of the 3D printed samples. The samples (5–10 mg) for TGA measurement were heated from 25 °C to 1000 °C at an increasing temperature rate of 10 °C/min under a nitrogen atmosphere.

#### 2.3.12. Thermal Conductivity Analysis

Thermal conductivity test was performed at room temperature with the assistance of a TC 3000 thermal conductivity tester (Xiatech Instrument Factory, Beijing, China) using a transient hot-wire method. The dimensions of each sample length × width were maintained at 30 × 10.00 mm.

## 3. Results and Discussion

### 3.1. Characterization of As-Prepared Different Temperature-Treated Anatase TNPs

**Figure 2:**
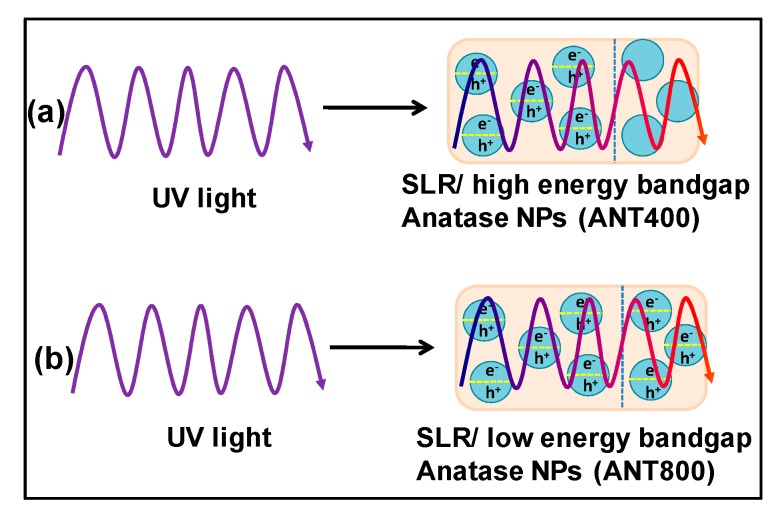
The key mechanism of photopolymerization process assisted by UV light penetration through the (**a**) stereolithography resin (SLR)/high energy bandgap semiconducting anatase nanoparticles (NPs) (ANT400) and (**b**) SLR/low energy bandgap semiconducting anatase NPs (ANT800). The energy/intensity of UV rays reduced (the violet color lines indicate higher intensity and red color lines indicates lower intensity) when it underwent deep penetration through the resin composites.

The as-prepared heat treated anatase TiO_2_ nanoparticle bandgap was analyzed with the assistance of diffusive reflectance spectroscopy (DRS), finding that, at 800°C, calcinaized anatase TiO_2_ showed the lowest bandgap among all the other heat-treated anatase TiO_2_. On the basis of the DRS analysis, Figure 2 demonstrates the proposed mechanism of the induced photopolymerization process assisted by UV irradiation. Figure 2 show that the energy or intensity of UV light decreased when its penetration depth increased through the SLA resin nanocomposites. The low bandgap semiconducting nanoparticles (here, ANT800) could generate more electron-hole pairs compared to high bandgap semiconducting NPs (here, ANT400) at high penetration depth where the energy of UV light was less. Thus, ANT800 can act as a catalyst to induce the polymerization through fast and strong bonding with monomers and oligomers present in the resin, owing to the improvement of the mechanical strength of the 3D printed polymer nanocomposites. The same phenomena was previously reported in the literature, expressing that the semiconducting nanoparticles are able to exhibit excellent mechanical properties due to their better UV light absorption capability and the possible production of free radicals as initiators by electron-hole pair generation, causing further propagation and high cross-linking of polymer chain [73,74,75].

**Figure 3:**
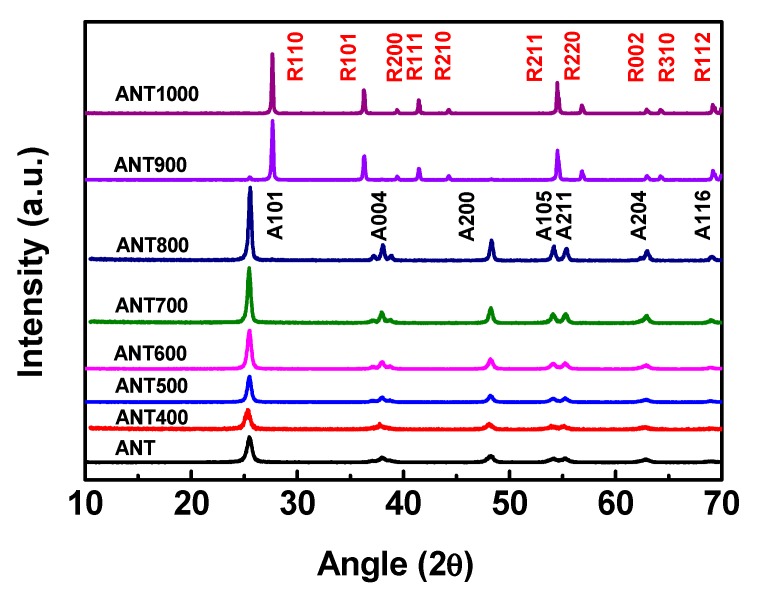
X-ray diffraction peaks of anatase TNPs annealed at different temperatures. The R and A represent the rutile and anatase phase, respectively, where the number followed by the R or A represents the crystal plane.

Herein, the correspondent crystalline phase of the different annealed anatase TNPs were performed by X-ray diffraction (XRD) and Raman spectroscopy analysis. The X-ray diffraction patterns of anatase TNPs are shown in Figure 3. The peaks found at 2θ angles of 27.5°, 36.2°, 39.3°, 41.4°, 44.3°, 54.5°, and 56.7° correspond to the diffractions from (110), (101), (200), (111), (210), (211), and (220) planes of rutile phase (matching with Joint Committee on Powder Diffraction Standards (JCPDS):73–1763) [76], which are indicated by R110, R101, R200, R111, R210, R210, R211, and R220 in Figure 3, respectively. The major peaks found at 25.6°, 38.1°, and 48.4° correspond to the diffractions from (101), (004), and (200) planes of phases of anatase (matching with JCPDS: 71-1166) [77], marked by A101, A004, and A200 in Figure 3, respectively. It can be found from Figure 3 that the anatase TNPs still remained in the anatase phase when heated from 400 to 800 °C, and the phase transition from anatase to rutile was noted at 900 °C, becoming 100% rutile phase at 1000 °C. However, at 800 °C, annealed anatase TNPs showed very high-intensity peaks compared with other TNPs, and it was confirmed that at 800 °C annealed anatase TiO_2_ exhibited remarkable crystalline structure, helping it to achieve better photopolymerization by strong cross-linking of nanoparticles within SLA resin during the 3D printing process. The same phenomena were further analyzed and confirmed by Raman and DRS analysis as well.

**Figure 4:**
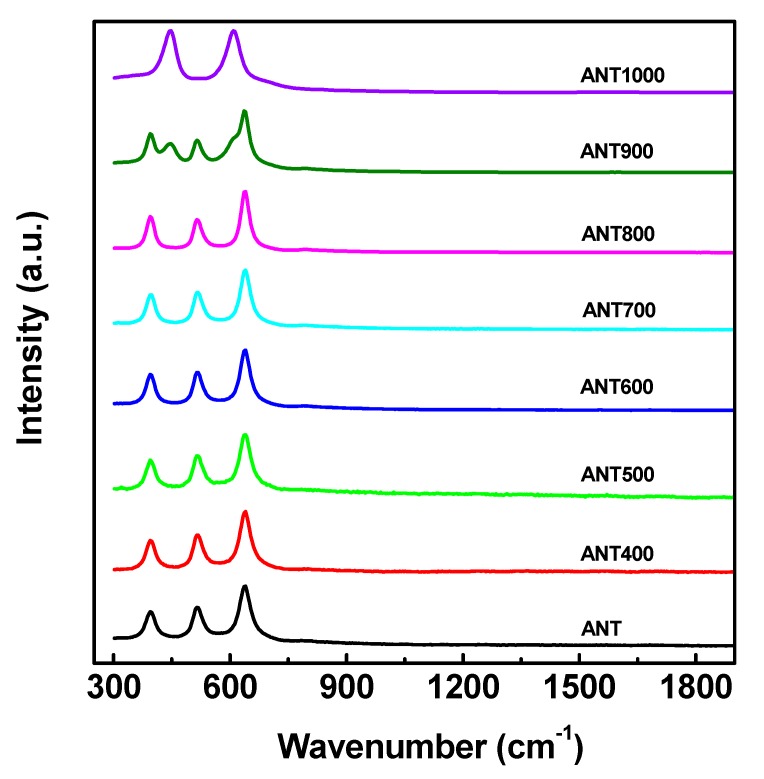
Raman spectrum of anatase TNPs annealed at different temperatures.

In addition to XRD, Raman spectroscopy was also used to investigate the structural identification of thermally treated anatase TNPs samples. The Raman spectrums of TiO_2_ polymorphs were unique enough, and were beneficial for distinguishing the various TiO_2_ phases. Figure 4 represents the Raman spectrum of as-prepared anatase TNPs annealed at different temperature samples. According to Figure 4, the Raman active modes, where the vibrational spectrum centered around 394.7, 514.6, and 639.8 cm^−1^, corresponded to the anatase phase, and the vibrational modes around 447.8 and 608.7 cm^−1^ represented the rutile phase [78]. The analysis of the Raman spectra of the sample annealed at 800 °C revealed high intensity, which was proof of high crystalline with a complete pure anatase phase. Further calcinations led to a gradual phase transformation from anatase to rutile. Both anatase and rutile vibrational modes were observed in the sample annealed at 900 °C, and after subsequent annealing at 1000 °C, the Raman spectra denoted that the phase had been completely transformed to rutile phase. The phenomena were noted and matched with the XRD analysis (Figure 3) study as well.

X-ray photoelectron spectroscopy (XPS) was extensively performed to characterize the composition of elements and electronic states of the samples. The resulting XPS survey spectrum is displayed in Supplementary Figure S2, where the presence of Ti and O elements are shown by the intensive peaks around 460 eV (Ti 2P_3/2_ at 459.37 eV and Ti 2P_1/2_ at 465.13 eV) and 531 eV (O–Ti at 530.60 eV and O–H at 531.98 eV) binding energy, respectively, in the TiO_2_ samples with significant contribution. Moreover, the deconvoluted XPS spectra of Ti and O are demonstrated in Figure S2b,c, respectively.

**Figure 5:**
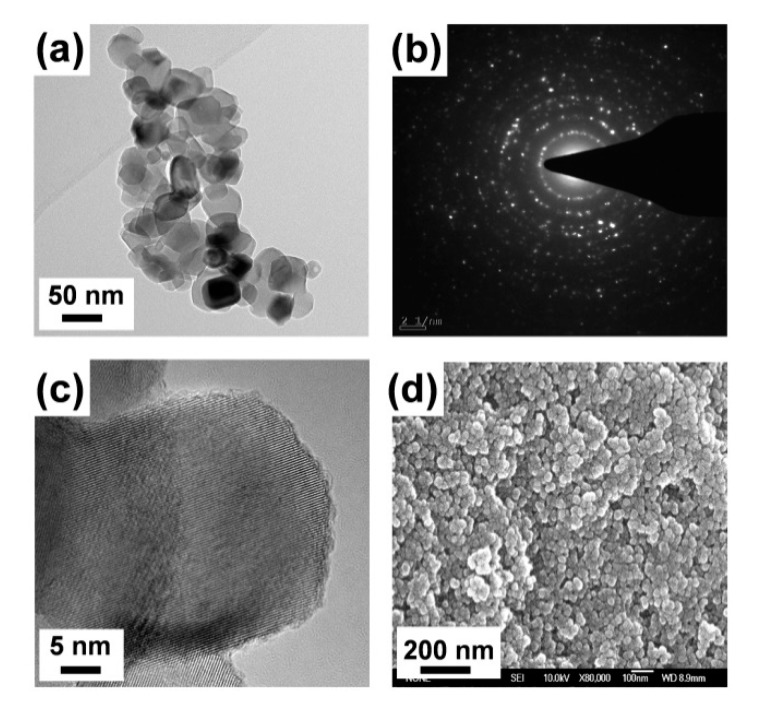
(**a**,**c**) High-resolution transmission electron microscopy (HRTEM) image of ANT800 NPs. (**b**) Selected area diffraction pattern (SAED) of ANT800 NPs. (**d**) Field emission scanning electron microscope (FESEM) image of ANT800 NPs.

The morphological study of as-treated ANT800 TNPs was performed by HRTEM and FESEM analysis, and the resulting images are displayed in Figure 5. Here, the ANT800 sample was mainly focused upon for morphological study, owing to its high crystalline phase and better mechanical performance among all other samples, which was discussed in the mechanical analysis of 3D printed samples as the best-incorporated material among the other annealed samples of anatase TNPs. The HRTEM images were taken after dispersing in pure ethanol and drop-casting on a copper grid. It can be observed from the images that the particles were found to be regular in shape (polyhedral) with a size distribution between 30–50 nm, and the common issues of agglomeration of nanoparticles were also found in the sample, which mean that it was not clear-cut in terms of finding a single particle. The inset in Figure 5a is the SAED pattern of the ANT800 NPs generated in HRTEM, which exhibited high crystalline structure and planes. The lattice fringes of ANT800 nanoparticles were further observed, as shown in Figure 5c. The resulting image of FESEM analysis of ANT800 is shown in Figure 5d. According to Figure 5d, the morphology of as-treated ANT800 was identified with the size of the TNPs being around 30–50 nm in terms of thickness, and slight agglomeration was noted.

To identify and understand the enhanced surface property and effects between the stereolithography resin (SLR) and incorporated anatase TNPs was performed by FTIR analysis, and the observed results are depicted in Supplementary Figure S3. All the FTIR analysis for this study was performed with as-ready vat slurry, before 3D printing. According to Figure S3, the observed FTIR results of ANT800, ANT800/KH570, SLR, and R/ANT800/KH570 revealed the better dispersion of surface-modified TNPs within the SLR matrix. The better dispersion was achieved by the surface treatment of anatase TNPs with the assistance of KH570 surfactant. In the FTIR patterns, the peaks around 678.82 cm^−1^ corresponded to the metal oxide peak (here, anatase TiO_2_ nanoparticles), which was present in every sample containing ANT800 [79]. It assured that the presence of anatase TNPs was significant in the solutions. The peaks at 2929.82 cm^−1^ belonged to C–H stretching, and the sharp peaks at 1723.56 cm^−1^ corresponded to C=O on the ester group of the SLA resin [80]. The quantity of surfactant KH570 was insignificant compared with SLA resin in detecting its wave number.

### 3.2. Characterization of SLA 3D Printed Samples

#### 3.2.1. Mechanical Properties

To understand the mechanical property enhancement of the 3D printed samples of neat SLR and annealed anatase, TNP-reinforced SLR (SLR/nanofillers) composites were performed by the assistance of universal testing machine and the resulting graphs are discussed in this subsection. Supplementary Figure S4 shows the stress–strain curves of the stated dog-bone-shaped 3D printed samples of neat SLR and SLR/nanofillers nanocomposites. Here, a constant 1% w/w loading of nanofillers was optimized and taken into account for further study into the SLA resin for all 3D printed samples, except neat SLR. For the 1% w/w nanofillers, optimization was performed and investigated by the aid of tensile strength analysis of various concentrations of untreated as-prepared anatase TiO_2_ nanoparticles (before calcinations of TiO_2_ NPs) incorporated with stereolithography resin, and the observed results are displayed in the Supplementary Information (Figure S5). The stress–strain curve from Supplementary Figure S5 shows that the 1% w/w loaded with the stereolithography sample showed a better tensile strength property among all the samples. Thus, on the basis of tensile strength analysis, the 1% w/w loading content of nanofillers with the photo resin was considered and further analyzed throughout the research work. For the samples annealed under different temperatures, the calcinations under the 800 °C (R/ANT800) sample showed better tensile strength (47.43 MPa) among all the samples, and was about 103% higher than the neat SLR printed sample (23.41 MPa). However, the enhancement of tensile strength was achieved through the incorporated ANT800 nanofillers, owing to its highly crystalline structure and a complete anatase phase [81]. Also, the ANT800 NPs possessed the lowest energy bandgap, which acted as a major role in boosting up the photopolymerization process, compared to the other different heat-treated anatase TNPs. Moreover, the R/ANT800 showed a low energy bandgap, a key factor in generating the electron-hole pairs rapidly, which could enhance the photopolymerization by a strong bonding with the monomers and oligomers present in the urethane-acrylate photo resin. Here, the introduced ANT800 nanoparticles helped to enhance the mechanical properties of 3D printed SLR resin through the achievement of better dispersion as well as the induced catalytic activity of the photopolymerization process. In addition to tensile strength, it can also be observed from Supplementary Figure S4 and Table 1 that the R/ANT800 had a better strain compared to all the other samples of nanofillers with SLA resin added.

**Figure 6:**
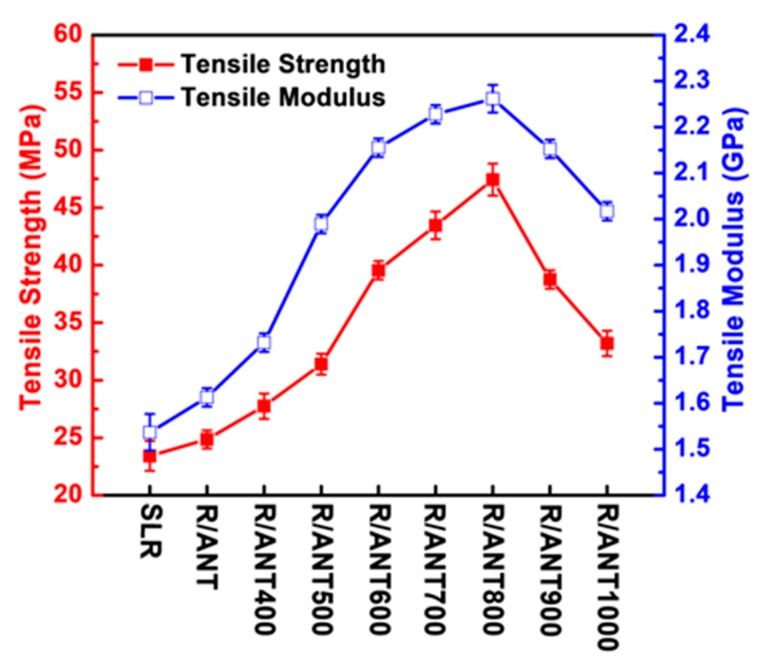
The complete dataset of tensile strength and elastic modulus of stereolithography (SLA) 3D printed samples prepared with the fillers of annealed anatase TNPs at different heat-treated temperatures.

For better understanding of the effect of reinforced nanofillers within the SLA resin on the mechanical properties, the complete dataset of elastic modulus and tensile strength is demonstrated in Figure 6 and Table 1. The correlation graph revealed that the tensile strength and elastic modulus of the 3D printed neat SLR was the lowest compared to the SLR/nanofillers nanocomposites. However, the SLR/nanofillers nanocomposites reinforced by the anatase TNPs without annealing (R/ANT) possessed the lowest tensile strength and elastic modulus, which indicated that the annealing treatment can effectively improve the mechanical properties of the SLR/nanofillers nanocomposites by the induced photopolymerization through the enhanced catalytic activity of annealed anatase TNPs. From the graphs, as the annealing temperature increased, the tensile strength and elastic modulus increased gradually up to 800 °C, which showed that, at 800 °C, annealed anatase TNPs were showcasing high crystalline structure and low energy bandgap, which led to better mechanical properties. In the case of increasing annealing temperature (more than 800 °C), the tensile strength and elastic modulus were noted as being remarkably decreased. Moreover, the 3D printed samples of R/ANT800 showed the efficient mechanical strength among all the other samples, with an improvement of 103% in tensile strength and 32% in elastic modulus, compared to the neat 3D printed SLR.

**Figure 7:**
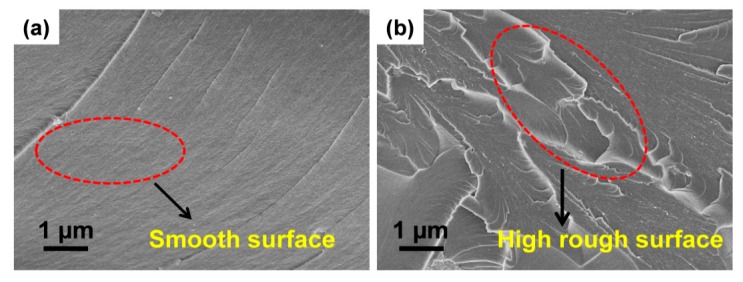
FESEM images of the fractured surface of tensile strength-tested 3D-printed samples: (**a**) neat SLR and (**b**) R/ANT800.

In addition to the better understanding of the mechanical reinforcement of neat SLR and SLR/nanofillers, the fractured surfaces of tensile-tested specimens were investigated by FESEM analysis. Figure 7 shows the FESEM images of the fractured portion of the tested tensile 3D printed samples of neat SLR and R/ANT800. It is vividly clear that the broken surfaces of printed SLR samples (Figure 7a) expressed higher micro-pores and smooth surfaces, which caused high brittleness, whereas the R/ANT800 samples (Figure 7b) possessed uniform dispersion and rough surfaces, which led to initiation of uniform cracking. For this reason, at 800 °C, the heat-treated anatase TiO_2_ nanoparticles achieved excellent dispersion within the SLR matrix, which may have been the key reason behind the enhancement of the mechanical and thermal properties of R/ANT800 nanocomposites.

**Figure 8:**
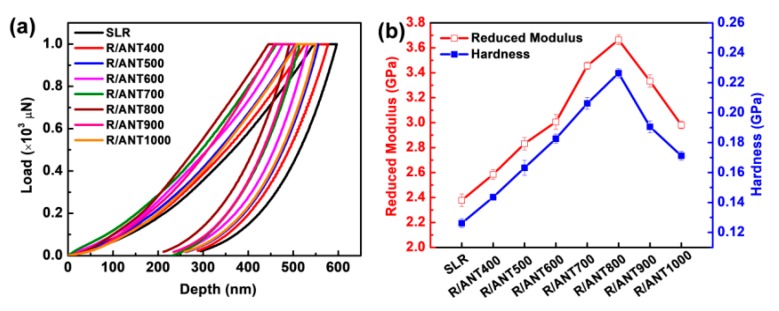
(**a**) Indentation load-depth curves for 3D printed samples of neat SLR and SLR/nanofillers composites. (**b**) The reduced modulus and hardness of 3D printed samples of SLR and SLR/nanofillers composites.

The analysis of the micro-mechanical properties (reduced modulus and hardness) of the neat SLR and SLR/nanofillers 3D printed specimens was performed with the assistance of nanoindentation analysis. Figure 8a, demonstrating the micro-mechanical analysis of the as-obtained nano-indentation results, shows that, with the addition of annealed anatase, TNPs were able to reduce the indentation depth remarkably. Conversely, with the loading of increasing annealing temperature of anatase TNPs, decreasing indentation depth of SLR/nanofillers was observed, especially at the loading of anatase TNPs annealed at 800 °C, showing shallow indentation depth, compared to all other annealed anatase, which was far better than the neat SLR sample. Moreover, Figure 8b, shows the extracted results of reduced modulus and hardness of the 3D printed samples of neat SLR and SLR/nanofillers. According to Figure 8b and Table 2, the reduced modulus and hardness of SLA resin gradually increased with an increasing annealing temperature of anatase TNPs. However, as the temperature of anatase TNPs increased up to 800 °C, expressing an increasing trend, a later sudden decrement of reduced modulus and hardness was noted, whereas the annealing temperature increased beyond 800 °C. The 3D printed sample of R/ANT800 showed a measured value of reduced modulus (3.66 GPa) and hardness (0.22 GPa), which were 54.4% and 69.2% higher than the neat SLR (2.37 GPa for reduced modulus and 0.13 GPa for hardness), respectively. As per our earlier discussion in this paper, the anatase TNP annealing temperature increased up to 800 °C, there were no phase changes, and high anatase crystalline phase was observed, but when the annealing temperature went beyond 800 °C, the treated anatase TiO_2_ started phase change from anatase to rutile. The obtained results clearly reveal that the anatase phases of TiO_2_ nanoparticles can remarkably increase the micro-mechanical properties of SLA resin matrix [81].

**Figure 9:**
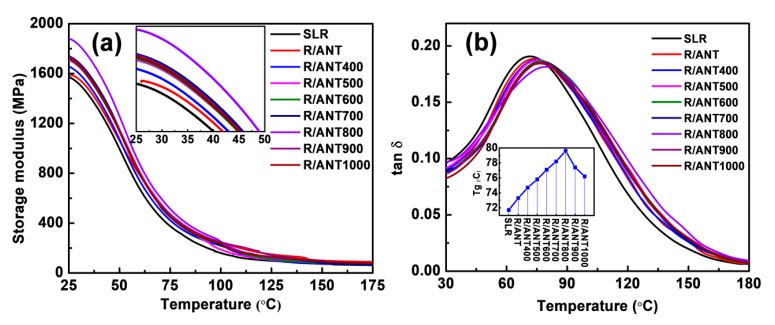
Thermo-mechanical analyses of neat SLR and SLR/nanofillers 3D printed samples. (**a**) Storage modulus and (**b**) tan δ curves. The inset in (**a**) shows the enlarged view of dynamic mechanical analysis (DMA) curves at a horizontal axis between 25 °C and 50 °C, and the inset in (**b**) shows the observed glass transition temperature (T_g_) of different samples from the tan δ curve.

The thermo-mechanical or dynamic mechanical analysis of neat SLR and SLR/nanofillers were analyzed through the assistance of Q800 TA instruments, and the observed results are displayed in Figure 9 and Table 3. The observed results showcase that the addition of anatase TNPs with SLR could enhance the storage modulus of the 3D printed structure compared to neat SLR. The gradual increases of storage modulus were noted, with the reinforcement of increasing annealed anatase TNPs up to 800 °C, and a decreased trend was observed when the reinforcement of annealed anatase TNPs was more than 800 °C (refer to the inset of Figure 9a). The sample R/ANT800 sustained the best storage modulus at 25 °C compared to all other samples, with the value of 1879.3 MPa, and it showed a level about 20% higher than the neat SLR printed sample of 1571.1 MPa. Figure 9b shows the tangent delta (tan δ) curves of the neat SLR and SLR/nanofillers 3D printed samples. The increased tan δ was observed at the loading of annealed anatase at 800 °C, and the decrement was observed beyond the loading of annealed anatase at 800 °C. The trend was noted as the same as the storage modulus. From the tan δ curves, it was demonstrated that the glass transition temperature (T_g_) of the 3D printed sample of R/ANT800 (79.62 °C) expressed the highest among all other samples, and could be noted as an elevated a difference of 7.9 °C compared to 3D printed neat SLR (71.72 °C) (refer to the inset graph in Figure 9b). It ensured that the R/ANT800 sample was thermally highly stable, compared to all the other samples.

**Figure 10:**
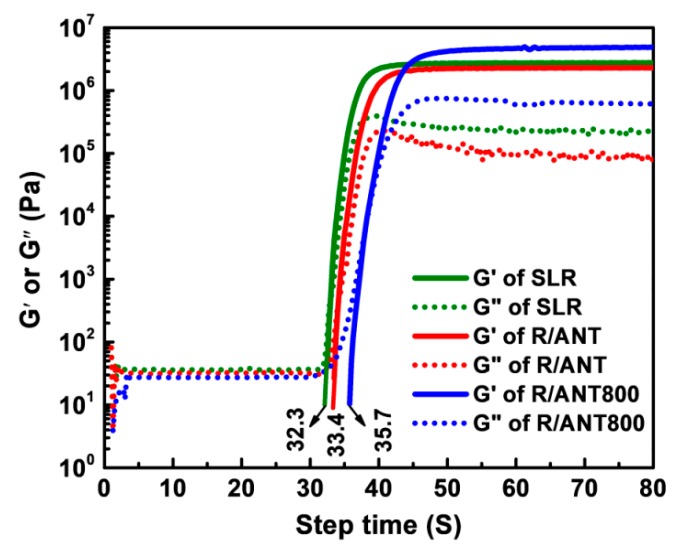
The effects of nanofillers on the kinetics of UV curing reaction. The solid and dotted lines represent storage modulus (G’) and loss modulus (G”) of the samples during the curing process, respectively.

A UV LED accessory of DHR-2 was performed to examine the curing behaviors of pristine SLR, SLR/ANT, and SLR/ANT800 nanocomposite matrix. The instrument can be recorded as the variations in storage modulus (G’) and loss modulus (G”) of the nanocomposite resin in the course of the curing process by the fast sampling process. Figure 10 demonstrates the curing behaviors of neat SLR and SLR containing 1% w/w of ANT NPs (without heat treatment) and 1% w/w of ANT800 NPs (heat-treated at 800 °C). For the UV-instigated photopolymerization and interconnection, the nanoparticle filler affected the gel time in two contradictory features: (1) the reinforcing effect of nanoparticles may endorse the interconnection between the monomers and oligomers to enhance the modulus of resin matrix, hence reducing the gel period; (2) the nanofillers may obstruct the light, hindering photopolymerization, and thus extending the gel period [82]. Figure 10 shows that the accumulation of anatase TNPs lengthened the gel time a slight amount, but the increased storage modulus was noted as compared to pristine SLR. Herein, the sample of R/ANT800 showed the best storage modulus (refer to the solid blue lines in Figure 10). However, its gel time was increased, initially owing to the nanoparticles hindering the UV light from penetrating far through the resin, though the excellent storage modulus was achieved through the outstanding dispersion and lower bandgap energy, which led to better mechanical property enhancement. In addition, from Figure 10, it can be observed that the SLR/nanofillers composites had a delay in the starting curing time, as compared to neat SLR in the photocuring reaction. This delay was due to the aggressive absorption of UV light by the photoinitiators or nanofillers composites [83].

#### 3.2.2. Thermal Properties

**Figure 11:**
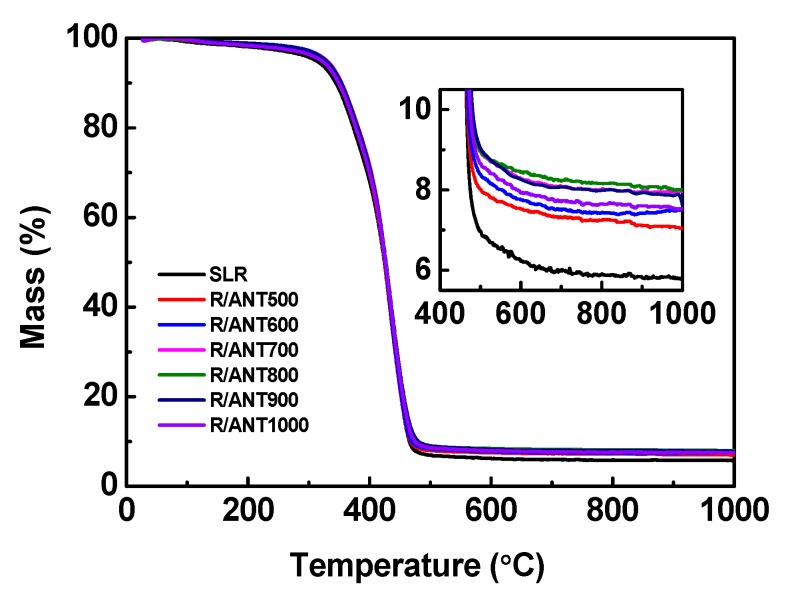
The thermal stability analysis for 3D printed samples of neat SLR and SLR/nanofillers 3D printed samples. The inset shows the enlarged view of the curves between 400 °C and 1000 °C to understand the final residual char values.

In order to describe the thermal stability of the neat SLR and SLR containing different annealed anatase TNPs, thermo gravimetric analysis was utilized, and the resulting graph is shown in Figure 11. With reference to Figure 11, the residual char weight of neat SLR (5.83%) is shown [84], and an increasing trend was observed during the addition of annealed anatase TNPs, especially in the loading of ANT800 (8.14%), which showed a better increment of residual char value, then decreasing for further annealed sample reinforcement. Earlier research reports that the incorporation of anatase TiO_2_ nanoparticles could possibly enhance the thermal stability of polymer matrixes [85]. The aforementioned interpretations propose that the advanced thermal stability with a lower decomposition rate was found in the sample R/ANT800, though at a higher temperature the defect solidity in high crystalline anatase phase was observed [86]. The key factor for the residual char enhancement was due to the lesser defect solidity with intensifying the calcinations temperature of anatase TNPs and enriched cross-linking between anatase TNPs and the SLR matrix at high temperature.

**Figure 12:**
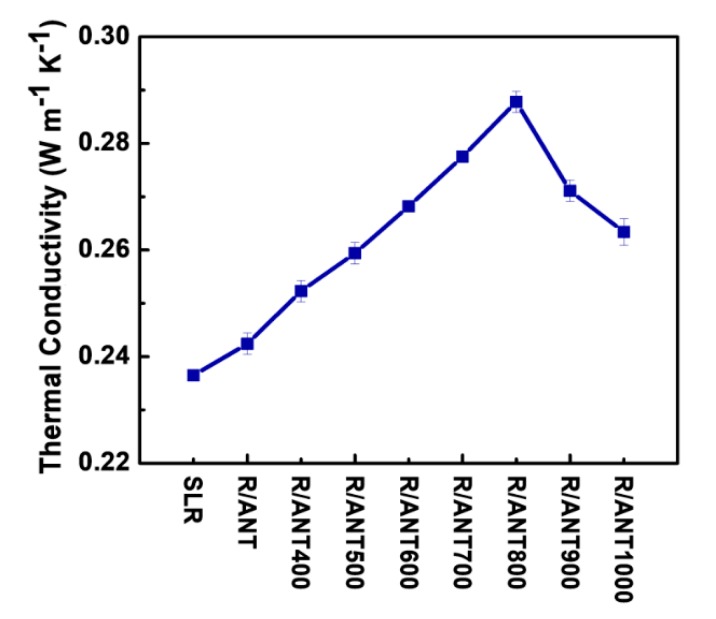
The thermal conductivity analysis of 3D printed samples of neat SLR and SLR/nanofillers.

Figure 12 demonstrates the thermal conductivity measurements of the SLR and SLR/nanofillers 3D printed samples. According to Figure 12, the addition of anatase TNPs with SLR could improve the thermal conductivity of a 3D printed structure. The better enhancement over thermal conductivity was distinguished with the incorporation of annealed anatase TNPs up to 800 °C, and a deprived trend was observed with annealed temperature beyond 800 °C. The enhanced thermal conductivity of R/ANT800 sample (0.2878 W/m K) was about 22% of the increment compared with the neat SLR (0.2365 W/m K). The better thermal conductivity of SLR was achieved through the incorporation of high crystalline anatase phase, which was attained at the annealing temperature of 800 °C. However, the earlier literature study reported that the addition of anatase TNPs to the polymer composites showed superior thermal conductivity compared with the addition of rutile or a mixture of both TNPs, owing to its small particle size and high crystalline structure of anatase TNPs [87]. Here, the smaller particle size (Figure 5a) and high crystalline anatase phase structure (Figure 3) of ANT 800 NPs led to achieving excellent dispersion within the SLR matrix, which helped to attain better improvement of thermal conductivity of the R/ANT800 sample.

**Figure 13:**
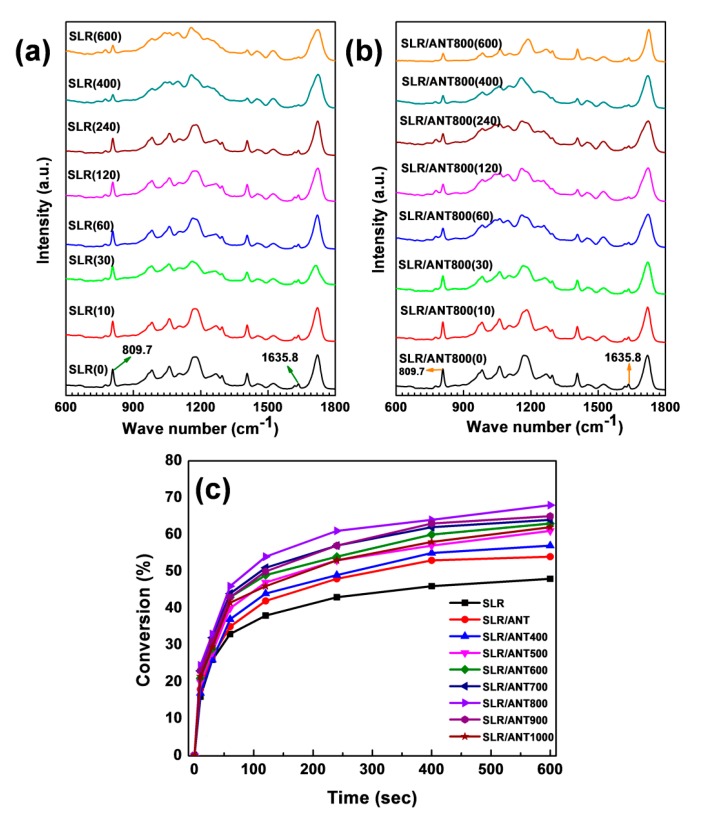
A real time FTIR analysis of photopolymerization of neat SLR and SLR/ANT800. (**a**) Neat SLR and (**b**) SLR/ANT800 nanofillers composite in different periods of UV irradiation time intervals starting from 0 s to 600 s. (**c**) Percent conversion graph of neat SLR and SLR/nanofillers.

The kinetic study of photo-polymerization reaction of both neat SLR resin and nanofillers incorporated SLR nanocomposites with the assistance of FTIR study, and the observed results are presented in Figure 13. As per the FTIR analysis, the free radical polymerization of multifunctional acrylates was pursued in real-time infrared spectroscopy by monitoring the decrease of the peak at 809.7 cm^−1^ of the acrylate double bond (C=C stretch) upon UV irradiation [88,89]. The analysis was performed on the basis of different time intervals to understand the photopolymerization process; initially the uncured vat slurry was investigated by FTIR, followed by UV curing at different time intervals such as 10s, 30s, 60s, 120s, 240s, 400s, and 600s. The neat SLA and SLR/ANT800 slurry analysis is presented in Figure 13, and all other SLR/nanofillers analyses such as SLR/ANT400, SLR/ANT500, SLR/ANT600, SLR/ANT700, SLR/ANT900, and SLR/ANT1000 are displayed in Supplementary Figure S6. The analysis was employed to determine the absorbance intensity of the peak at 809.7 cm^−1^, which is related to acrylate double bond. From the FTIR analysis, the percentage conversion versus time curves was plotted to understand the photopolymerization reactions based on the C=C conversion ratio occurring in different time periods. Figure 13 shows that the conversion rates of the photopolymerization reactions were enhanced by the addition of filler content. Figure 13 express that the SLR/ANT800 composite showed a better C=C conversion rate of photopolymerization among all other SLR/nanofillers composites. The percentage conversion of SLR/ANT800 was reached at 24.5% at the 10 s point, where percent conversion of SLR was only 10.2%. When the time reached to 600 s, the percent conversion of SLR/ANT800 was 68.1%, where the SLR showed only 47.8%. The percentage of conversion is expressed by the following equation [90]:

(1) $$\%\;\mathrm{Conversion}\;=\frac{A_{0}+{\;A}_{t}}{A_{0}}\times 100\%$$

where A_0_ are the absorbances of double bonds at 809.7 cm^−1^ before irradiation, and A_*t*_ are the absorbance of double bonds at 809.7 cm^−1^ after t seconds irradiation time.

### 3.3. Mechanical and Thermal Enhancement Mechanism of SLR/Nanofiller 3D Printed Samples

**Figure 14:**
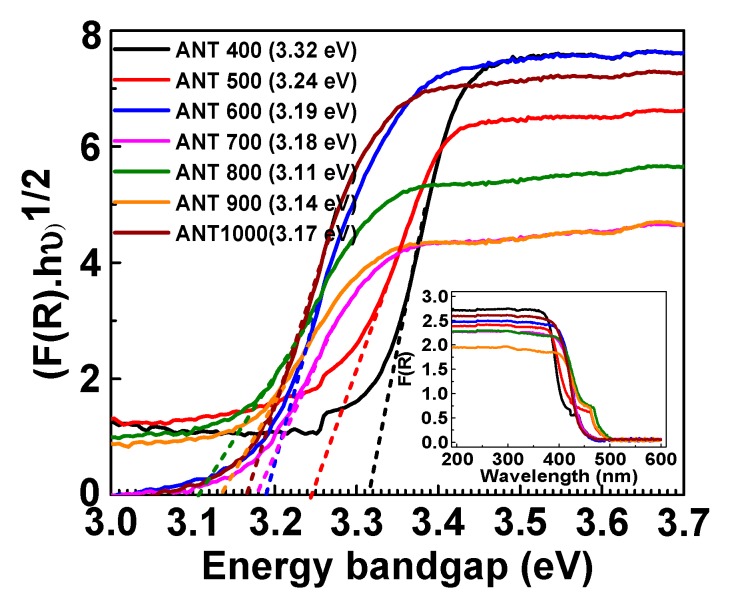
Tauc’s plot for an indirect bandgap model (Kubelka-Munk function) and UV-visible diffusive reflectance spectra (inset) of annealed anatase TNPs. The dash-dot lines represent line tangent towards the point, where the line intersected the horizontal energy axis (bandgap). The observed bandgap of controllably annealed anatase TNPs is mentioned in brackets, followed by the sample name.

To better understand the mechanical and thermal enhancement of incorporated anatase TNPs within the SLR matrix, diffusive reflectance spectroscopy was performed, and the curves for energy bandgap analysis using the Kubelka-Munk equation were further plotted in Figure 14. According to the analysis, the Kubelka-Munk function F(R) allowed the UV absorbance of the semiconducting anatase TNPs to be estimated from its reflectance (R) according to the following equation:

F(R) = (1 − R)^2^/2R (2)

The bandgap measured by the slope of the Tauc’s curve linearly extended towards the point where the line intersected the horizontal energy axis by means of the subsequent Tauc’s plot appearance when supposing that the anatase TNPs were indirect bandgap semiconductors.

(F(R)·hν)^1/2^ = A (hν − E_g_) (3)

Where A, h, and v are the constant, the Planck constant, and the frequency, respectively [91,92]. From this graph (refer to Figure 14), it was precisely measured that the bandgap of anatase TNPs reduced when increasing the annealed temperature up to 800 °C, and was raised to above 800 °C. The ANT800 NPs had the lowest bandgap (3.11 eV) among all samples. The lowest energy bandgap would help the anatase semiconducting TNPs rapidly and immensely generate the electron-hole pairs under UV light, which triggers the vast and strong polymerization throughout the photo resin [93].

The UV light irradiation on the surface of semiconducting anatase TNPs can undergo photo-excitation, which would induce the generation of electrons in conduction band (CB) and holes in valence band (VB), as represented in Equation (4) [94]:

TiO_2_ + hν→ TiO_2_ (e^−^_CB_ + h^+^_VB_). (4)

The as-produced holes in VB can oxidize acrylic monomers, leading to the generation of free radicals (refer to Equation (4)), which can promote the initiation of a polymerization reaction (refer to Equation (6)).

h^+^_VB_ + A → A ^•^+ H^+^, (5)

A ^•^+ A → P. (6)

Moreover, the photo-reduction process by CB electrons can undergo the reaction correspondingly.

e^−^_CB_ + A →A^•−^ (7)

A^•−^ + H^+^→ H A^•^ (8)

The combination of electrons in CB with protons would cause the formation of atomic hydrogen followed by H A^•^ radicals, which would help initiate the polymerization reaction.

e^−^_CB_ + H^+^→ H ^•^, (9)

A + H ^•^→ H A^•^, (10)

where, A, A^•^, and P represents the acrylate based monomer, monomer radical, and polymer, respectively.

**Figure 15:**
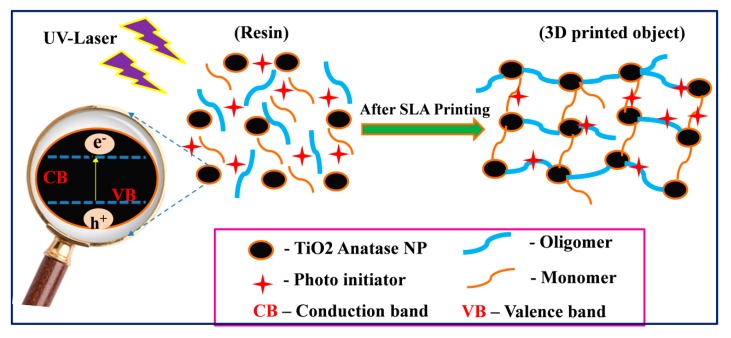
A schematic illustration of photopolymerization of photo resin with semiconducting anatase TNPs under UV light for the SLA 3D printing.

**Figure 16:**
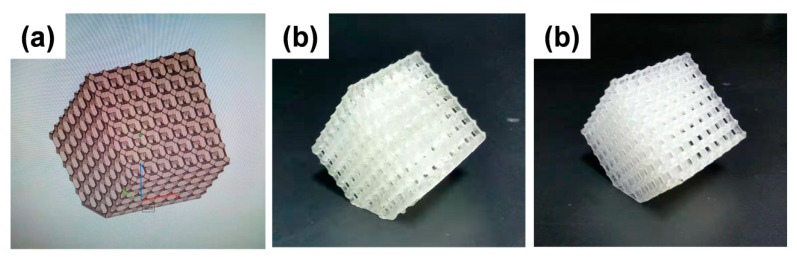
(**a**) 3D model designed by computer-aided design (CAD) software, (**b**) and (**c**) are 3D printed objects using neat SLR and R/ANT800 composite samples by SLA 3D printer apparatus, respectively.

It was evidently confirmed by the above mechanical and thermal analysis that the sample R/ANT800 showed an outstanding enhancement of mechanical and thermal properties. The excellent property enhancement was achieved for R/ANT800, meaning that the ANT800 NPs having a highly crystalline anatase phase and lowest energy bandgap were able to generate a rapid enormous number of electron-hole pairs, which can lead to the strong cross-linking between monomers and oligomers in the R/ANT800 sample. The detailed schematic illustration of incorporated annealed anatase TiO_2_ induced photopolymerization proposed mechanism has been depicted in the Figure 15. The pictorial representation of the enhanced bonding mechanism of photopolymerization by the incorporation of anatase TNPs with the SLA resin matrixes was depicted in the supplementary Figure S7. Figure 16 shows digital photography of the 3D printed objects using neat SLR and R/ANT800 composite samples by SLA 3D printer.

## 4. Conclusions

The semiconducting anatase TNPs were synthesized using sol-gel method and annealed at different temperatures from 400 °C to 1000 °C. It was shown from the analysis of XRD and Raman spectra that the as-prepared annealed anatase TNPs still remained in the anatase phase when heated from 400 °C to 800 °C, and that the phase transition from anatase to rutile appeared at 900 °C, becoming completely transformed to rutile phase at 1000 °C. The reinforcement of annealed anatase TNPs in the SLA resin showed remarkable enhancement in the mechanical and thermal properties of the SLA 3D printed objects. In particular, the loading of ANT800 (R/ANT800) performed with better mechanical strength (103% enhancement as compared with neat SLR) and high thermal stability (elevated to 7.6 °C). In addition, enhanced thermal conductivity and residual char yield of about 22% and 8.14% were respectively achieved. The high surface area and lower bandgap energy of the anatase TNPs helped to achieve good dispersion, high chance to bind with monomers, and also permitting UV light to penetrate at maximum depth through the photo resin. The highly crystalline anatase TNPs could generate more electron-hole pairs compared to rutile TNPs because the anatase TNPs had lower energy bandgap as compared with rutile TNPs. The lowest bandgap of ANT800 (3.11 eV) triggered the generation of enormous electron-hole pairs and facilitated sturdy and rapid polymerization within resin, which could lead to superior mechanical and thermal properties of 3D printed samples. Altogether, our findings can be a new path to draw more interest in the presently projected fields.

## Figures and Tables

**Table 1:** Extracted results of tensile strength, tensile modulus, and % strain of 3D printed samples of pure SLR and SLR/nanofillers composites.

Sample	Tensile Strength (MPa)	Tensile Modulus (GPa)	Strain (%)
Neat SLR	23.42 ± 1.3	1.53 ± 0.04	4.55 ± 0.4
R/ANT	24.87 ± 0.8	1.61 ± 0.02	1.79 ± 0.3
R/ANT400	27.76 ± 1.1	1.73 ± 0.02	1.98 ± 0.2
R/ANT500	31.40 ± 0.9	1.98 ± 0.02	2.22 ± 0.4
R/ANT600	39.55 ± 0.8	2.15 ± 0.02	2.42 ± 0.3
R/ANT700	43.46 ± 1.2	2.22 ± 0.02	2.63 ± 0.2
R/ANT800	47.43 ± 1.4	2.26 ± 0.03	3.06 ± 0.4
R/ANT900	38.73 ± 0.8	2.15 ± 0.02	2.59 ± 0.2
R/ANT1000	33.20 ± 1.1	2.01 ± 0.02	2.27 ± 0.2

**Table 2:** Results of 3D printed samples of pure SLR and SLR/nanofillers nanocomposites from the nanoindentation test.

Sample	Max Indentation Depth (nm)	Reduced Modulus (GPa)	Hardness (GPa)
Neat SLR	596.60 ± 32.2	2.3776 ± 0.01	0.1262 ± 0.003
R/ANT400	577.35 ± 26.3	2.5856 ± 0.02	0.1436 ± 0.002
R/ANT500	555.55 ± 20.2	2.8318 ± 0.02	0.1632 ± 0.005
R/ANT600	532.97 ± 18.4	3.0062 ± 0.02	0.1826 ± 0.003
R/ANT700	513.56 ± 30.3	3.4552 ± 0.01	0.2062 ± 0.004
R/ANT800	490.87 ± 24.2	3.6645 ± 0.02	0.2264 ± 0.003
R/ANT900	507.74 ± 26.3	3.3328 ± 0.03	0.1906 ± 0.004
R/ANT1000	550.60 ± 31.2	2.9814 ± 0.01	0.1712 ± 0.003

**Table 3:** The glass transition temperature and storage modulus values of neat SLR and SLR with the fillers of annealed anatase TNPs at different calcinations temperatures.

Sample	Storage 30 °C	Modulus (MPa) 100 °C	Tan δ Peak Height	T_g_ (°C)
Neat SLR	1515.72	160.63	0.191	71.7
R/ANT	1555.24	188.18	0.189	73.3
R/ANT400	1601.18	220.65	0.188	74.7
R/ANT500	1652.33	230.02	0.187	75.8
R/ANT600	1659.90	238.82	0/186	77.1
R/ANT700	1684.91	245.08	0.185	78.2
R/ANT800	1827.31	252.08	0.181	79.6
R/ANT900	1675.70	247.39	0.184	77.4
R/ANT1000	1665.93	236.84	0.185	76.2

## Supplementary Materials

The following are available online at https://www.mdpi.com/2079-4991/10/1/79/s1, Figure S1: A general scheme of an SLA 3D printing apparatus. Figure S2: FTIR spectra of ANT800, ANT800/KH570, SLR, and R/ANT800/KH570. Figure S3: Stress–strain curves for the 3D printed samples of neat SLR, and nanofillers-reinforced SLR nanocomposites with 1% w/w loading content of anatase TNPs annealed under different temperatures from 400 °C to 1000 °C. The inset shows the model of 3D printed sample prepared for tensile strength test. Figure S4: Stress-strain curves for the 3D printed samples of neat SLR, and nanofillers reinforced SLR nanocomposites with 1% w/w loading content of anatase TNPs annealed under different temperatures from 400 °C to 1000 °C. The inset shows the model of 3D printed sample prepared for tensile strength test. Figure S5: (a) Stress-strain curves for the 3D printed samples of neat SLR and different % w/w loading of anatase TiO_2_ before calcinations (ANT) nanofillers in the SLR. (b) The comparative analysis of tensile strength investigation of neat SLR and SLR/ANT with different weight percentage. Figure S6: FTIR spectra for analyzing kinetics of photopolymerization reactions of (a) SLR/ANT (b) SLR/ANT400, (c) SLR/ANT500, (d) SLR/ANT600, (e) SLR/ANT700, (f) SLR/ANT900 and (g) SLR/ANT1000 nanocomposites under the UV exposure in different time intervals starting from 0 to 600 s. Figure S7: The pictorial representation of polymerization bonding mechanism of incorporated anatase TiO_2_ and acrylate monomers.
