# Supplementary material for: Enhanced Mechanical and Thermal Properties of Stereolithography 3D Printed Structures by the Effects of Incorporated Controllably Annealed Anatase TiO2 Nanoparticles
Source: Nanomaterials (Basel). 2020 Jan 1;10(1):79. doi: 10.3390/nano10010079 (PMC7022956; doi:10.3390/nano10010079)
Supplement: Supplementary file 1 [file nanomaterials-10-00079-s001.pdf]

Supplementary Materials

Enhanced Mechanical and Thermal Properties of Stereolithography 3D Printed Structures by the Effects of Incorporated Controllably Annealed Anatase TiO_2 Nanoparticles

Suhail Mubarak [1,2], Duraisami Dhamodharan [1,2], Nidhin Divakaran [1,2], Manoj B. Kale [1,2], T. Senthil [3], Lixin Wu [1,*] and Jianlei Wang [1,*]

[1] CAS Key Laboratory of Design and Assembly of Functional Nanostructures, Fujian Key Laboratory of Nanomaterials, Fujian Institute of Research on the Structure of Matter, Chinese Academy of Sciences, Fuzhou 350002, China; suhail@fjirsm.ac.cn (S.M.); duraisamidhamodharan@fjirsm.ac.cn (D.D.); nidhin@fjirsm.ac.cn (N.D.); manojkale@fjirsm.ac.cn (M.B.K.)

[2] University of Chinese Academy of Sciences, Beijing 100049, China

[3] Advanced Research School for Technology and Product Simulation, Central Institute of Plastics Engineering and Technology, Chennai 600032, India; tsenthilsci@gmail.com

* Correspondence: lxwu@fjirsm.ac.cn (L.W.); jlwang@fjirsm.ac.cn (J.W.)

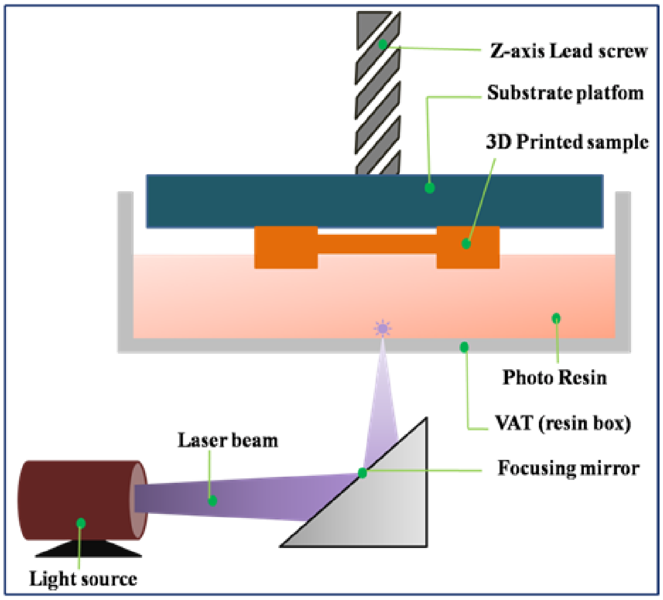

Figure S1 A general Scheme of an SLA 3D printing apparatus.

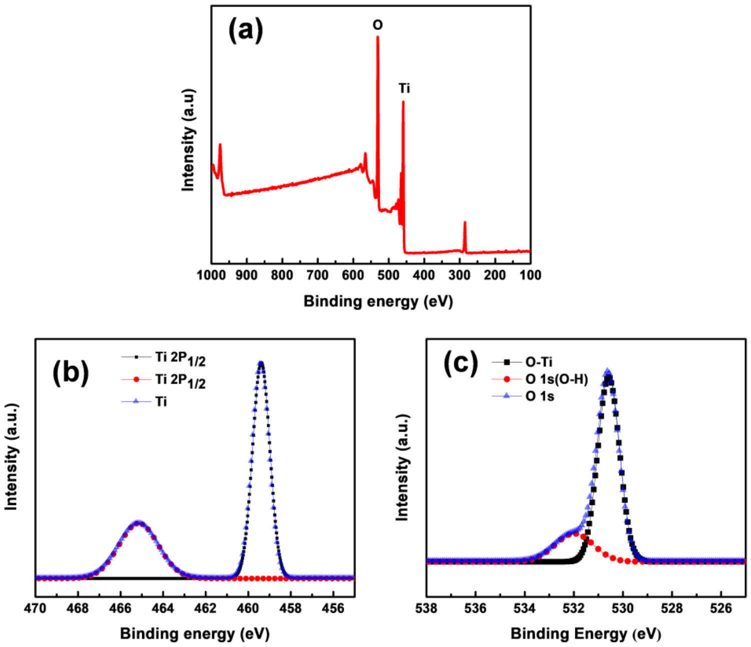

Figure S2 XPS spectra of anatase TNPs: (a) survey spectrum of TiO_2,(b) Deconvoluted XPS spectra of Ti, and (c)Deconvoluted XPS spectra of O.

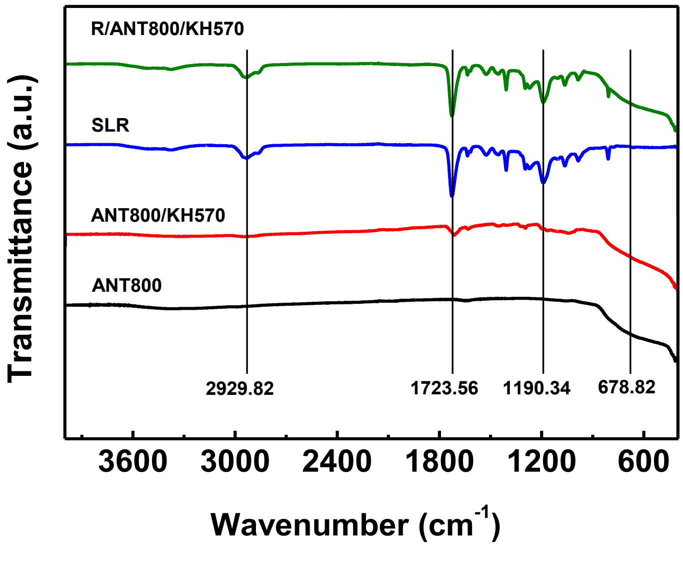

Figure S3 FTIR spectrums of ANT800, ANT800/KH570, SLR, and R/ANT800/KH570

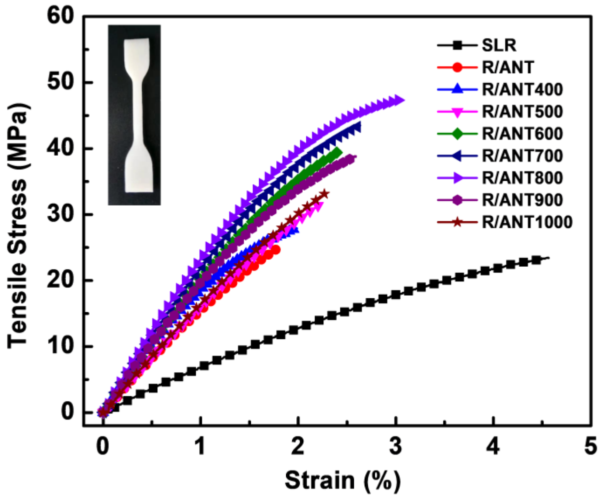

Figure S4 Stress-strain curves for the 3D printed samples of neat SLR, and nanofillers reinforced SLR nanocomposites with 1% w/w loading content of anatase TNPs annealed under different temperatures from 400 °C to 1000 °C. The inset shows the model of 3D printed sample prepared for tensile strength test.

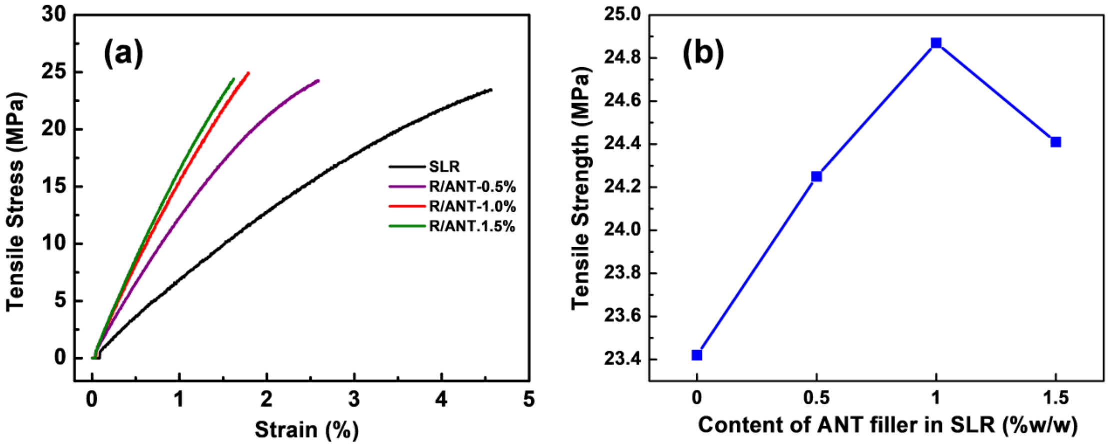

Figure S5(a) Stress-strain curves for the 3D printed samples of neat SLR and different % w/w loading of anatase TiO_2 before calcinations (ANT) nanofillers in the SLR. (b) The comparative analysis of tensile strength investigation of neat SLR and SLR/ANT with different weight percentage.

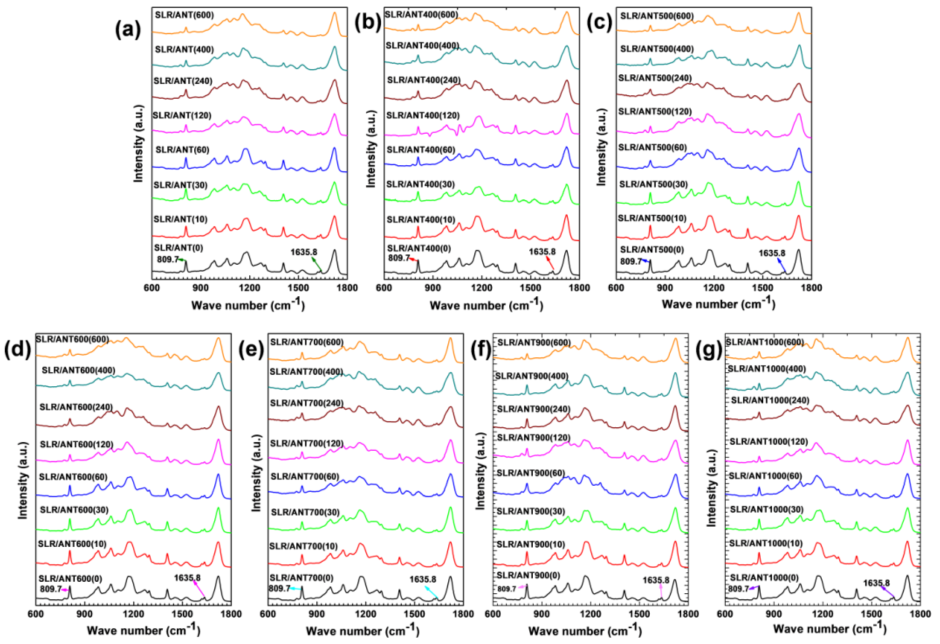

Figure S6 FTIR spectra for analyzing kinetics of photopolymerization reactions of (a) SLR/ANT (b) SLR/ANT400, (c) SLR/ANT500, (d) SLR/ANT600, (e) SLR/ANT700, (f) SLR/ANT900 and (g) SLR/ANT1000 nanocomposites under the UV exposure in different time intervals starting from 0 to 600 seconds.

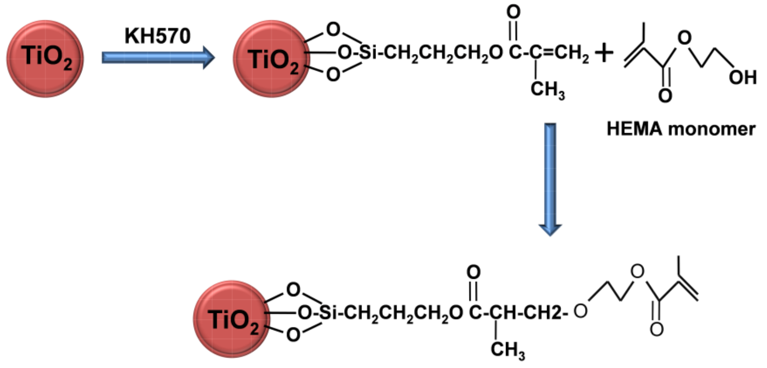

Figure S7 The pictorial representation of polymerization bonding mechanism of incorporated anatase TiO_2 and acrylate monomers.
